# Dynamic spectral signatures of mirror movements in the sensorimotor functional connectivity network of patients with Kallmann syndrome

**DOI:** 10.3389/fnins.2022.971809

**Published:** 2022-08-25

**Authors:** Federica Di Nardo, Renzo Manara, Antonietta Canna, Francesca Trojsi, Gianluca Velletrani, Antonio Agostino Sinisi, Mario Cirillo, Gioacchino Tedeschi, Fabrizio Esposito

**Affiliations:** ^1^Department of Advanced Medical and Surgical Sciences, University of Campania “Luigi Vanvitelli,” Naples, Italy; ^2^Department of Neuroscience, University of Padova, Padova, Italy; ^3^Department of Medicine, Surgery and Dentistry, University of Salerno, Salerno, Italy

**Keywords:** Kallmann syndrome, mirror movements, dynamic functional connectivity, sensorimotor network, K-means, connectivity states

## Abstract

In Kallmann syndrome (KS), the peculiar phenomenon of bimanual synkinesis or mirror movement (MM) has been associated with a spectral shift, from lower to higher frequencies, of the resting-state fMRI signal of the large-scale sensorimotor brain network (SMN). To possibly determine whether a similar frequency specificity exists across different functional connectivity SMN states, and to capture spontaneous transitions between them, we investigated the dynamic spectral changes of the SMN functional connectivity in KS patients with and without MM symptom. Brain MRI data were acquired at 3 Tesla in 39 KS patients (32 without MM, KSMM-, seven with MM, KSMM+) and 26 age- and sex-matched healthy control (HC) individuals. The imaging protocol included 20-min rs-fMRI scans enabling detailed spectro-temporal analyses of large-scale functional connectivity brain networks. Group independent component analysis was used to extract the SMN. A sliding window approach was used to extract the dynamic spectral power of the SMN functional connectivity within the canonical physiological frequency range of slow rs-fMRI signal fluctuations (0.01–0.25 Hz). K-means clustering was used to determine (and count) the most recurrent dynamic states of the SMN and detect the number of transitions between them. Two most recurrent states were identified, for which the spectral power peaked at a relatively lower (state 1) and higher (state 2) frequency. Compared to KS patients without MM and HC subjects, the SMN of KS patients with MM displayed significantly larger spectral power changes in the slow 3 canonical sub-band (0.073–0.198 Hz) and significantly fewer transitions between state 1 (less recurrent) and state 2 (more recurrent). These findings demonstrate that the presence of MM in KS patients is associated with reduced spontaneous transitions of the SMN between dynamic functional connectivity states and a higher recurrence and an increased spectral power change of the high-frequency state. These results provide novel information about the large-scale brain functional dynamics that could help to understand the pathologic mechanisms of bimanual synkinesis in KS syndrome and, potentially, other neurological disorders where MM may also occur.

## Introduction

The occurrence of involuntary hand movements that mirror a voluntary movement of the contralateral hand, a neurological symptom referred to as bimanual synkinesis or mirror movement (MM), is considered physiological only during childhood (up to the age of 10) (Beaulé et al., 2012). However, it could persist during adulthood in congenital conditions like Kallmann syndrome (KS). An imbalance of the developing brain motor circuit has been suggested as a possible cause for reduced suppression of involuntary contralateral hand movements (Mayston et al., 1997; Farmer et al., 2004).

In a previous resting-state fMRI (rs-fMRI) multi-center study on KS (Manara et al., 2018), the presence of the MM symptom was found to be associated with abnormal spectral changes in the static functional connectivity (sFC) of the large-scale sensorimotor network (SMN). More specifically, a relatively lower contribution of the so called “slow-5” frequency band (0.01–0.027 Hz) together with a relatively higher contribution of the so called “slow-3” frequency band (0.073–0.198 Hz), has been reported from the spectral analysis of the spontaneous fluctuations of the SMN time-course, in KS patients with MM (MM+) compared to KS patients without MM (MM−). These effects were further characterized in terms of imbalance between cortical-cortical functional connectivity (more prevalent in the slow five band) and cortical-subcortical functional connectivity (more prevalent in the slow three band) to explain the reduced suppression of involuntary contra-lateral hand movements systematically occurring in MM+ patients when voluntary hand movement is requested.

However, as the human brain is a highly dynamic system, the resting-state functional connectivity has been largely proven to be temporally varying (Chang and Glover, 2010). That is, temporal fluctuations in the functional connectivity of a large-scale functional network, such as the SMN, may also reflect dynamic changes in the corresponding domain-specific functional connectivity with possible non-stationary switching between two or more discrete recurrent patterns or states. This has posed the natural question about whether the previously highlighted spectral signature of the MM symptom in KS patients constitutes an intrinsic stationary feature of the SMN functional connectivity, most likely secondary to abnormal anatomical structures within the motor circuitry, or, rather, is itself subject to dynamic temporal fluctuations between recurrent states, provided that a sufficiently long period of time (e.g., 20 min or more) is used for the observation (Hindriks et al., 2016). In other words, it is not known whether the functional connectivity of SMN can exhibit more than one recurrent (patho)physiological states, whose dynamic features, such as, e.g., the different contributions to the dynamic spectral changes in the canonical frequency bands, appear under- or over-represented in KS subjects manifesting the MM symptom.

As previous results were obtained with a purely static FC analytic approach, i.e., observing rs-fMRI signals from a large-scale network over a typical, yet short, period of 5–10 min, the current study aimed at verifying if a dynamic FC (dFC) analysis, and more specifically a dynamic spectral power analysis of the network-specific amplitude of low-frequency fluctuations, would also disclose similar characteristic dFC features in KS patients with MM.

The most common and straightforward way to investigate dFC is using windowed FC (Hutchinson et al., 2013), which consists of calculating a given FC measure over consecutive and overlapping segments of the rs-fMRI time-course data (e.g., 1–2 min), thus providing a time series of FC values, which can subsequently be used to assess dynamic fluctuations in FC over a substantially longer rs-fMRI session (e.g., 20–30 min). Such dFC analysis would also allow to identify the presence of recurrent spectral patterns for a given large-scale brain network, i.e., two (or more) dFC states with different spectral characteristics of the network time-course of activity, between pairs of which the same network spontaneously fluctuates over time.

To the best of our knowledge, this would be the first rs-fMRI study investigating, with such a spectral dFC approach, the possible spectral dFC correlates of MM in KS patients, thus potentially gathering new insights into the more dynamic aspects of the cerebral motor circuitry derangement associated with the clinical manifestation of the bimanual synkinesis.

## Materials and methods

### Subjects and experimental design

Thirty-nine patients with KS (38 male, mean age ± SD 32.53 ± 11.61 and one female, age 13) were enrolled for this study. All patients met the diagnostic criteria for KS, based on clinical findings and smell analysis (hypogonadotropic hypogonadism and hypo/anosmia). The study was approved in accordance with the requirements of the local Ethical Committee at the University Hospital “San Giovanni Di Dio e Ruggi D’Aragona” of Salerno and written informed consent was obtained from patients or their parents. All KS patients underwent a complete physical and neurological examination including the evaluation of handedness and the evaluation of MM according to Woods and Teuber (1978) criteria. In particular MM were scored as follows: “0” absent; “1” barely discernible but repetitive movements; “2” either slight but sustained movement or stronger but briefer repetitive movement; “3” strong and sustained repetitive movement; “4” movement equal to that observed in the intended hand: this phenomenon may be prevalent on the right hand or on the left one. In this way, subjects were divided into two groups: KS patients with MM (KSMM+, mean age ± SD: 34.86 ± 16.94) and KS patients without MM (KSMM–, mean age ± SD: 31.41 ± 10.73). Thus, we have 7 KSMM + and 32 KSMM–. We also scanned 26 healthy age-matched control subjects without MM.

Table 1 reports the full demographical and clinical profile of all KS patients including type of olfactory dysfunction (anosmia/hyposmia), handedness, clinical MRI abnormalities, grade (0–4) of MM separately for right and left hand and with side preference of MM.

**TABLE 1:** Patients’ clinical profile.

Pat.#	Olfactory status	bOb aplasia/hypoplasia	MM (R)	MM (L)	MM (R + L)	MM (R vs. L)
1	Anosmia	Yes	No MM	No MM	No MM	n.a.
2	Anosmia	Yes	No MM	No MM	No MM	n.a.
3	Anosmia	Yes	No MM	No MM	No MM	n.a.
4	Anosmia	Yes	No MM	No MM	No MM	n.a.
5	Anosmia	Yes	No MM	No MM	No MM	n.a.
6	Anosmia	Yes	No MM	No MM	No MM	n.a.
7	Anosmia	Yes	No MM	No MM	No MM	n.a.
8	Anosmia	Yes	No MM	No MM	No MM	n.a.
9	Anosmia	Yes	No MM	No MM	No MM	n.a.
10	Anosmia	Yes	No MM	No MM	No MM	n.a.
11	Anosmia	Yes	No MM	No MM	No MM	n.a.
12	Anosmia	Yes	No MM	No MM	No MM	n.a.
13	Anosmia	Yes	No MM	No MM	No MM	n.a.
14	Anosmia	Yes	No MM	No MM	No MM	n.a.
15	Anosmia	Yes	No MM	No MM	No MM	n.a.
16	Anosmia	Yes	No MM	No MM	No MM	n.a.
17	Anosmia	Yes	No MM	No MM	No MM	n.a.
18	Anosmia	Yes	No MM	No MM	No MM	n.a.
19	Anosmia	Yes	No MM	No MM	No MM	n.a.
20	Anosmia	Yes	No MM	No MM	No MM	n.a.
21	Anosmia	Yes	No MM	No MM	No MM	n.a.
22	Anosmia	Yes	No MM	No MM	No MM	n.a.
23	Anosmia	Yes	No MM	No MM	No MM	n.a.
24	Anosmia	Yes	No MM	No MM	No MM	n.a.
25	Anosmia	Yes	No MM	No MM	No MM	n.a.
26	Anosmia	Yes	No MM	No MM	No MM	n.a.
27	Anosmia	Yes	No MM	No MM	No MM	n.a.
28	Anosmia	Yes	No MM	No MM	No MM	n.a.
29	Anosmia	Yes	No MM	No MM	No MM	n.a.
30	Anosmia	Yes	No MM	No MM	No MM	n.a.
31	Anosmia	Yes	No MM	No MM	No MM	n.a.
32	Anosmia	Yes	No MM	No MM	No MM	n.a.
33	Anosmia	Yes	4	3	7	Right
34	Anosmia	Yes	2	1	3	Right
35	Anosmia	Yes	2	3	5	Left
36	Anosmia	Yes	2	0	2	Right
37	Anosmia	Yes	3	3	6	No prev.
38	Anosmia	Yes	3	3	6	No prev.
39	Anosmia	Yes	3	2	5	Right

L, left; R, right; MM, grade of mirror movements according to Woods and Teuber criteria; no prev., no R vs. L prevalence; bOBs, bilateral olfactory bulbs.

### Magnetic resonance imaging acquisition

MRI image data sets were acquired on a 3T MRI scanner (MAGNETOM Skyra, Siemens, Erlangen Germany) equipped with a 20-channel radiofrequency receive head coil. The imaging protocol consists of a volumetric anatomical scan, followed by resting-state fMRI scan.

The anatomical scans were performed with a 3D T1-weighted magnetization prepared rapid gradient echo sequence (MPRAGE) with TR/TE: 2400/2.25 ms; resolution: 1 mm; matrix size: 256 × 256. Resting-state fMRI scans consisted of 1,800 volumes and 44 slices, performed with a gradient-echo echo planar imaging (GRE-EPI) with a multiband factor of 4 (Feinberg et al., 2010; Moeller et al., 2010; Xu et al., 2013), TR/TE: 662/30 ms, matrix size: 64 × 64; voxel size: 3 × 3 × 3 mm^3^, direction of phase encoding acquisition anterior-posterior. The same GRE-EPI series was repeated two more times with only five dynamic scans and opposite phase encoding directions (anterior-posterior, posterior-anterior) for the purpose to correct GRE-EPI image distortion (Andersson et al., 2003; Smith et al., 2004). Each scanning acquisition was about 25 min long: 20 min for functional imaging and 5 min for anatomical imaging. During the functional scan, subjects were asked to simply stay motionless and awake.

### Functional magnetic resonance imaging data preprocessing

Each individual resting-state fMRI time series was first corrected for the different slice scan acquisition times (*via* cubic spline interpolation) and for rigid head motion effects (*via* realignment of all volumes to the first) using BrainVoyager QX (Brain Innovation, Maastricht, Netherlands^1^). Subsequently, the image time series were first exported to NIFTI format for geometrical distortion correction *via* the TOPUP tool of FSL (Andersson et al., 2003; Smith et al., 2004). Then, the subsequent preprocessing steps were performed on distortion-corrected NIFTI images using the Data Processing Assistant for Resting-State fMRI (DPARSF) (Yan and Yu-Feng, 2010),^2^ which is based on Statistical Parametric Mapping (SPM)^3^ and on the toolbox for Data Processing and Analysis of Brain Imaging (DPABI) (Yan et al., 2016).^4^ The alignment of the first volume of each subject resting-state fMRI series to the corresponding anatomical 3D-T1w image was implemented with affine transformation; then, all T1w images were normalized to the MNI space with the non-linear diffeomorphic DARTEL approach (Ashburner, 2007); lastly, the coregistered functional data were normalized to the MNI space with the transformations obtained during the DARTEL procedure.

To reduce the residual effects of head motion, as well as the effects of respiratory and cardiac signals, second-order motion and physiological nuisance correction were performed using a linear regression approach: the regression model included 24 motion-related predictors (Friston et al., 1996), with six head motion parameter time-series, their first-order derivatives and the 12 corresponding squared parameter time-series; the mean time-courses from a white matter mask and a cerebrospinal fluid mask (as obtained from 3D-T1w spatial segmentation) were added as two additional predictors. In order to account for residual motion-related spikes, an additional spike-related regressor was created from the frame wise displacement time-series, i.e., a predictor with a value of 1 at the time points of each detected spike and a value of 0 elsewhere (Lemieux et al., 2007; Satterthwaite et al., 2013). Finally, the image time series were band-pass filtered between 0.01 and 0.5 Hz and spatially smoothed with an isotropic 6-mm full width at half maximum (FWHM) Gaussian kernel.

To minimize the potential effects of head motion and possibly exclude subjects exhibiting excessive amounts of motion, we applied severe inclusion criteria: the six estimated head motion parameters (three translation and three rotation) were considered and subjects exhibiting head translations >3 mm and/or head rotations >3 degrees were excluded from consecutive analyses. Then, the mean frame wise displacement value (FD) was estimated as an additional measure of total instantaneous head motion (Power et al., 2012; Kim et al., 2017) and the percentage of spike-corrupted volumes in each time-series was calculated. Potential spike-corrupted volumes were identified where the FD value exceeded a threshold of 0.5 mm; at this stage, subjects for whom the percentage of corrupted volumes exceeded 50% in the scan were also excluded from the analyses.

### Functional magnetic resonance imaging data analysis

Data were decomposed into functional networks using a group-level spatial ICA as implemented in the Group ICA (GICA) of functional MRI Toolbox (GIFT)^5^ (Calhoun et al., 2001; Correa et al., 2005). The number of components to be extracted was estimated from the resting-state fMRI data using the minimum description length (MDL) criterion (Li et al., 2007a,b) applied to the concatenated data set of patients and healthy controls, ensuring the same number of components for all the patients and healthy controls. Prior to data reduction, voxel-wise variance normalization was applied to the time course of each voxel (Beckmann and Smith, 2004; Allen et al., 2010). Then, two data reduction steps of Principal Component Analysis (PCA) were performed (subject-specific and group-level) using the expectation maximization algorithm and the independent components were extracted using the Infomax algorithm (Bell and Sejnowski, 1995; Esposito et al., 2002) and repeated 20 times through ICASSO (Himberg et al., 2004); finally, the GICA back reconstruction algorithm (Calhoun et al., 2001; Erhardt et al., 2011) provided participant’s spatial maps and their corresponding time courses.

For the dFC analysis, a sliding window approach was performed through custom scripts written in MATLAB R2021a (The MathWorks Inc., Natick, MA, United States^6^) to explore time-varying changes of FC within the individual network components during functional MRI acquisitions. More in details, three hundred and thirty-one tapered sliding windows were obtained by segmenting the time-course of each subject into windows 150 volumes (150 TR = 99.3 s) with a step of five volumes (5 TR = 3.31 s). In fact, a window size between 30 s and 1 min was shown to be a reasonable choice for capturing brain dynamics (Shirer et al., 2012; Allen et al., 2014; Damaraju et al., 2014; Rashid et al., 2014; de Lacy et al., 2017). Using the time-series data of all selected independent component pairs within each window, a pairwise covariance matrix was calculated.

Spectral power information was obtained using the time-course of activity corresponding to the selected individual independent component. Following Zuo et al. (2010), we further subdivided the relative contribution of each independent component time-course spectrum to the whole detectable frequency range into four separate bands (Table 2). The cross-spectrum was calculated in MATLAB over the entire low-frequency range of interest (0.01–0.25 Hz) *via* the cross-spectrogram function by specifying a Hamming sliding window. As a result, a dynamic spectrum connectivity (DSC) matrix was obtained, representing the changes in the amplitude of the time-course of network activity as a function of time over the entire duration of the scan. For the purposes of this study, only the SMN component was selected and considered. However, as the SMN connectivity may variably include the contribution from the subcortical structures, an additional region of interest (ROI) based analysis of the dynamic functional connectivity was performed on the fMRI signals from the basal ganglia and thalamus. Namely, using the Harvard–Oxford subcortical structural atlas (with 2 mm resolution) distributed with the FMRIB Software Library, we anatomically subdivided the basal ganglia into caudate, putamen and pallidum (De Micco et al., 2019) and downsampled the resulting mask to the size of fMRI data (3 mm).

**TABLE 2:** Frequency range for the four canonical bands.

Bands	Frequency interval (Hz)
Slow-5	0.01–0.027
Slow-4	0.027–0.073
Slow-3	0.073–0.198
Slow-2	0.198–0.25

To assess recurrent dFC patterns over time, a k-means clustering algorithm was performed to the windowed DSC matrix (Allen et al., 2014; Fu et al., 2018, 2019; Espinoza et al., 2019; Schumacher et al., 2019). The k-means clustering was applied twice: first, to find the optimal number of clusters *via* silhouette criterion, and second, to perform clustering analysis with the obtained cluster optimal number (Rousseeuw, 1987; Kim et al., 2017; Fiorenzato et al., 2019). The frequency of each state was estimated for each subject as the proportion of windows assigned to a state (cluster). The number of transitions between different states was also calculated for each subject. Then, a one-way ANOVA analysis of both frequency and transitions was performed considering the group as a between-subject factor with three levels: KSMM+, KSMM- and healthy controls.

## Results

No significant differences were found between HC subjects and KS patients and between the two KS subgroups (MM−, MM+) in age and gender. None of the enrolled subjects were excluded from the analysis as all passed the inclusion criteria used for the inter- and intra-voxel residual motion effects.

**FIGURE 1:**
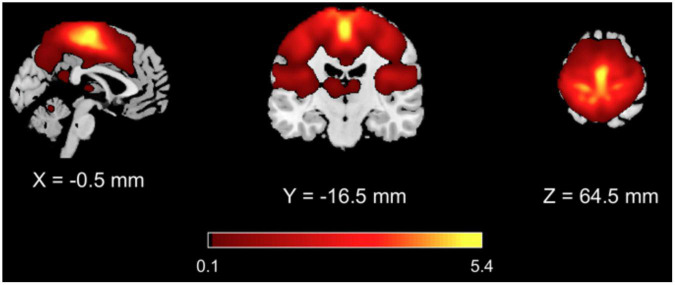
Results of GICA analysis. The SMN component was selected as the one with highest z values in the primary and supplementary motor areas and in the primary and secondary sensory areas.

From the GICA analysis, 13 components were extracted among which the SMN component was selected as the one whose spatial map exhibited highest z values bilaterally in the primary and supplementary motor areas and in the primary and secondary sensory cortices (Figure 1 and Table 3).

**TABLE 3:** Coordinates of the three peaks obtained from the sensorimotor network map.

Regions	MNI coordinates (x, y, z)
Supplementary motor area	−1, −20, 67
Left primary motor cortex	−18, −35, 70
Right primary motor cortex	17, −35, 74

**FIGURE 2:**
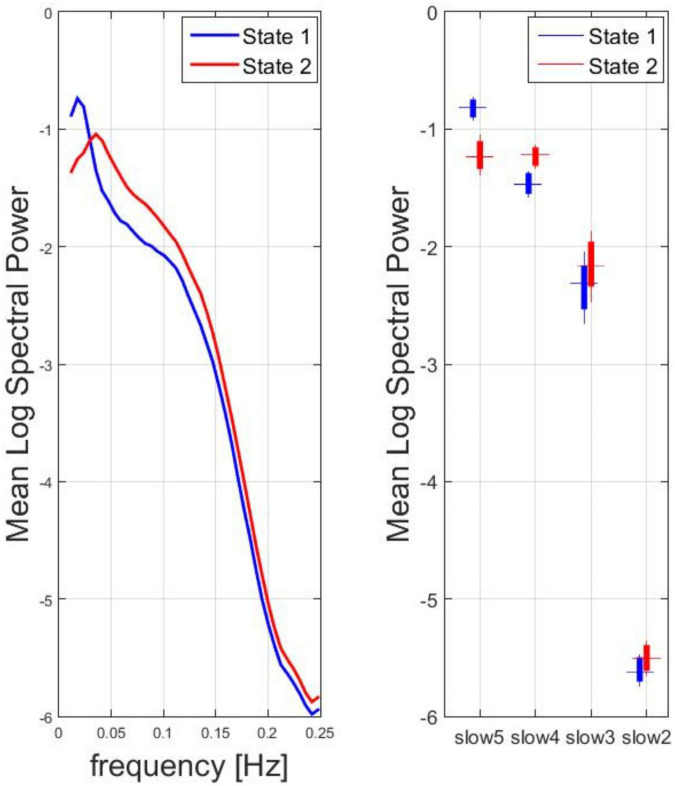
Spectral analysis for two clusters of most recurrent dFC states: State 1, State 2. Left: for the two states, the mean logarithm of the spectral power (vs. frequency) in the range between 0.01 and 0.25 Hz was calculated. According to the peak frequency, state 1 was descriptively identified as a low-frequency dFC state, whereas state 2 was descriptively identified as a high-frequency dFC state. Right: Box plot of the mean log spectral power in the four canonical frequency bands across all subjects.

The SMN dynamic spectral power data from all subjects and time windows were used in the k-means clustering, resulting in two clusters of most recurrent dFC states: state 1, state 2. For the two states, the mean spectral power (vs. frequency) in the range between 0.01 and 0.25 Hz and the box plot of the mean spectral power in the four canonical frequency bands across all subjects are displayed in Figure 2. According to the peak frequency of the mean spectral power of each state, state 1 was descriptively identified as a low-frequency dFC state, whereas state 2 was descriptively identified as a high-frequency dFC state. Indeed, across all subjects, the mean spectral power was significantly higher for state 1 vs. state 2 in the lowest frequency canonical band (slow 5: one-sample paired *t*-test, *p* < 0.0001) whereas the opposite held true for the other canonical bands (slow 4 and slow 3: one-sample paired *t*-test, *p* < 0.0001; slow 2: one-sample *t*-test, *p* < 0.01).

**FIGURE 3:**
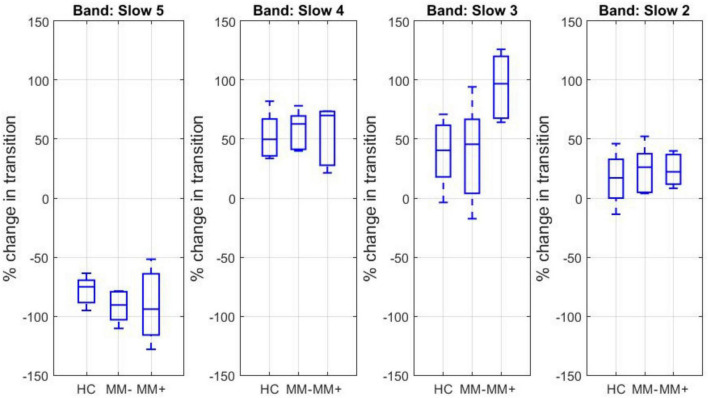
Boxplots of the percent signal change in the mean log spectral power associated with transitions between two dFC states for each canonical frequency band and each experimental group.

For each canonical frequency band and each experimental group (HC, MM−, MM+), the percent signal change in the mean spectral power associated in average with any transition between two dFC states across two adjacent time windows was estimated. The corresponding boxplots are displayed in Figure 3.

The percent spectral power change associated with the transitions from state 1 to state 2 was negative for slow 5 and positive for slow 4, slow 3, and slow 2. The percent spectral power change for slow 3 band was significantly increased in the group of MM + patients (and about double in size) compared to both MM− patients (two-sample *t*-test, *p* = 0.0013) and HC subjects (two-sample *t*-test, *p* = 0.0015).

**FIGURE 4:**
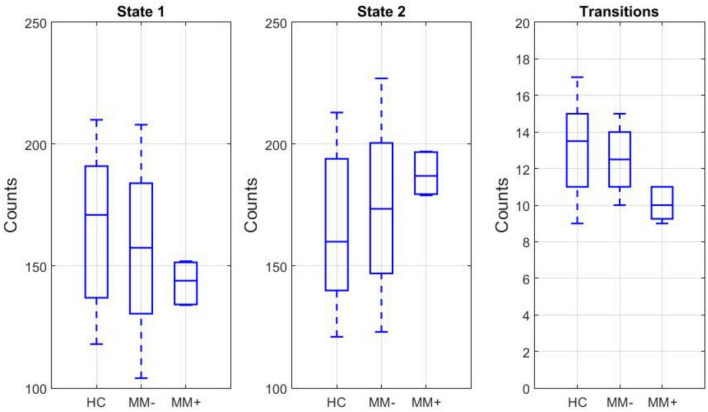
Left and middle: box plots of the frequency of occurrence in the state 1 and state 2 for each experimental group. Right: box plot of the frequency of state transitions between the two states.

For each subject, both the frequency of occurrence of each state, i.e., independently of the mean spectral power in predefined canonical bands, and the frequency of state transitions between the two states were counted. The box plots of these counts across experimental groups are displayed in the Figure 4. While the number (count) of time windows associated with each state did not significantly differ between groups or between states, there was a significant group-by-state interaction (2-way ANOVA, *p* = 0.04). Moreover, even if *post hoc t*-test revealed no significant differences in the counts between subgroups (in both states), there was a significant reduction in the number of transitions between states in MM+ patients compared to both HC subjects (two-sample *t*-test: *p* = 0.001) and MM− patients (two-sample *t*-test, *p* = 0.013).

From the basal ganglia and thalamus ROI analysis, for each frequency band, each group (HC, MM−, MM+) and each ROI, the percent signal change in the mean spectral power associated in average with any transition between two dFC states across two adjacent time windows was also estimated but no significant differences were revealed. Finally, for each subject and each ROI, both the frequency of occurrence of each state and the frequency of state transitions between the two states were counted. One-way ANOVA analysis from basal ganglia and thalamus ROI analyses revealed no significant differences in the number of transitions between groups. In each state, there were no significant differences in term of the frequency of the occurrences (count of time windows associated with each state). Moreover, in the same regions, there were no significant group-by-state interactions (2-way ANOVA).

## Discussion

This study explored the dynamic spectral changes of the intrinsic functional connectivity of the large-scale sensori-motor brain network in KS patients and HC subjects, demonstrating that KS patients presenting at the clinical examination with the phenomenon of bimanual synkinesis (or MM) may also exhibit different spontaneous fluctuations of the spectral content of SMN component over a 20 min period of observation between two most recurrent oscillatory states.

First, we extracted a common group ICA component for the SMN of the whole group of KS patients and HC subjects with the purpose of extracting the most general spatial pattern characterizing the whole-brain co-activation of the most functionally connected motor regions. Starting from the SMN group component, the subject-specific SMN time-courses of activity were submitted to a sliding-window spectral analysis and a cluster analysis of the spectral power identified two stable and recurrent dFC states: a low-frequency state (state 1) and a high-frequency state (state 2).

Many previous studies have supported the notion that neural oscillations supporting the functional connectivity of the human brain can exhibit frequency-dependent properties, even within the small range of slow rs-fMRI signal fluctuations (Zuo et al., 2010). In general, relatively higher frequency neuronal oscillations (e.g., in the gamma band in the EEG signal) are restricted to a relatively smaller spatial scale, whereas long-range neuronal communications are supported by slower oscillations (e.g., in delta band of the EEG signal) (Buzsáki and Draguhn, 2004). Accordingly, a general theory for brain oscillations, regardless of the scale of spatio-temporal observation, would prescribe that the longer the range of functional connectivity (among remote brain regions), the lower the frequency of the functional connectivity signal emerging from the integration of brain functions, the physiological rationale being that remote regions with different functional specialization most likely oscillate at different frequencies (Wang et al., 2016) and several rs-fMRI studies have shown how the strength of large-scale networks decreases when the frequency increases (Wu et al., 2008; Gohel and Biswal, 2015; Li et al., 2015). Particularly, in the context of large-scale brain functional networks, the functional processes supporting long-range connections among spatially distributed cortical regions normally operate in a lower frequency band compared to those supporting short-range connections within more spatially compact subcortical regions (Buzsáki and Draguhn, 2004). In line with this notion, we had previously observed how slow 5 fluctuations of rs-fMRI signals were more characteristic of a cortical-cortical static functional connectivity, whereas slow 4 and slow 3 were more characteristic of a cortical-subcortical static functional connectivity [see, e.g., Esposito et al. (2013) and Manara et al. (2018)]. Here, for the first time, we were able to demonstrate that at least two distinct (i.e., stable and recurrent) dynamic functional connectivity states may co-exist in the dynamic functional connectivity of the SMN in terms of a different contribution of relatively lower- and higher-frequency oscillatory components. This would imply that (i) there are shorter windows of time (∼1.5 min) where the slower cortical-cortical oscillations would prevail in the SMN functional connectivity against the faster subcortical-cortical oscillations and that (ii) the SMN network would spontaneously (and randomly) fluctuate between such periods, the balance between the occurrences of these two states becoming an interesting new element for the neuroimaging assessment of the motor circuitry functional integrity. In the more specific context of the MM symptom, here we found that, not only the relative spectral change in the switching between these two states was in average significantly increased in the slow 3 band in KS patients with MM (compared to HC subjects and KS patients without MM), but also the number of such transitions was significantly reduced in those patients that therefore appear to persist in the high-frequency dFC state for much longer time than needed or expected. This demonstrates how MM can be seen as a clinical manifestation of a neural deficit in dynamic flexibility of the SMN.

Importantly, the dynamic spectral analysis of the SMN did not show significant differences between KSMM− patients and controls, indicating that the dynamic spectral shifts observed in the motor circuit of KS MM+ patients may not (primarily) depend on the general KS condition itself or some specific (KS-related) hormone or treatment differences between KS patients and controls. In addition, the absence of regional spectral differences in subcortical ROIs (when taken in isolation from the SMN) may suggest that the dynamic functional changes primarily depend upon on long-range connectivity changes affecting the resting-state cortical activity. More specifically, following up our previous line of interpretation (Manara et al., 2018), we could hypothesize that the spontaneous synchronization of cortical motor areas is abnormally attracted toward a high-frequency state due to an abnormal functioning of the cortical-subcortical loop that controls voluntary movements. On the other hand, the lack of difference between MM+ patients and healthy controls in the functional connectivity of basal ganglia and thalamus was at least unexpected as the role of the interhemispheric control in the case of unilateral movements is well-known. Thus, we cannot exclude that this null finding was due to the lack of statistical power implied by the small size of the MM + group (including only three patients with right unilateral MM and only one patient with left unilateral MM) and anyway future studies (involving a larger sample of MM+ patients) are needed to address the relation between the changes observed here in the SMN functional connectivity and the left-right coupling of the resting-state oscillations across cortical and subcortical homotopic regions.

The analysis of time-varying brain activity and connectivity using rs-fMRI has become an important topic of ongoing neuroscience discussions. Indeed, significant changes in the temporal dynamics of brain network connectivity (both in terms of configuration and synchronization) have been reported in different neurological diseases, thereby some researchers have hypothesized that this type of analysis might eventually provide some important biomarkers of disease [see, e.g., Hutchinson et al. (2013) and Damaraju et al. (2014)].

A crucial point of this study is that KS is a genetic disease in which we can see some functional aspects of a neurological disease, including the presence of MMs, that have originally suggested an involvement of the cerebral motor circuit. However, in KS, structural data from previous neuroimaging studies have provided conflicting results [see, e.g., Krams et al. (1997, 1999), Leinsinger et al. (1997); Koenigkam-Santos et al. (2008)Koenigkam-Santos et al. (2010), and Manara et al. (2015)]. For example, abnormal values of the magnetization transfer ratio at level of the pyramidal decussation were observed in KS patients independently of the presence of MM (Koenigkam-Santos et al., 2010), but diffusion tensor imaging studies did not reveal structural changes of the cortical-spinal tract in KS patients with or without MM (Manara et al., 2014, 2015). At the cortical level, KS patients with MM showed significant cortical thinning in small regions known to be involved in the voluntary hand motor control and bilateral volume decrease of the globus pallidum, compared with KS patients without MM, thus suggesting a complex readjustment of the motor circuitry associated with bimanual synkinesis (Manara et al., 2015). Our results would thus confirm in a newly designed rs-fMRI study on new KS patients, the observations of a previous study (Manara et al., 2018) based a static functional connectivity approach, in which the analysis also revealed a significant group by frequency interaction pointing to a frequency shift in the spectral content in KS patients. However, as the present study was purposefully designed to perform a dynamic functional connectivity analysis, we were here able to pinpoint a more finally detailed aspect of KSMM+ functional connectivity: namely that these patients tend to switch from a lower frequency state of brain connectivity to a higher frequency with significantly greater facility than healthy controls and KSMM− patients and consequently tend to spend more time in this high frequency state.

As mentioned above, a dFC approach similar to the one presented here has been previously employed in other psychiatric and neurological diseases, including Schizophrenia, Parkinson’s Disease, Alzheimer’s Disease, autism or Huntington disease. In these pathologies, k-means clustering procedures have usually shown transitions among more than two brain networks states and the changes between these transitions were mostly related to cognitive (Rashid et al., 2014; Fiorenzato et al., 2019; Schumacher et al., 2019) or motor (Kim et al., 2017) impairments. On the other hand, most KS patients are cognitively intact and only a small percentage of them develop bimanual synkinesis, which therefore characterize a very rare condition. Consequently, the neural underpinnings of MM phenomenon remain unclear, albeit the dynamic point of view on the functional connectivity addressed here seems promising with respect to the need of better addressing this aspect of the pathology. Of course, larger-sample studies, possibly integrating dFC from other networks or regions remain needed to better elucidate the pathogenic mechanism of MM in KS and in other congenital or acquired conditions, as well as in neurodegenerative diseases.

In conclusion, we have performed, to our knowledge, the first dFC analysis of the SMN, determining two discrete frequency-specific oscillatory states, in KS patients with and without MM. Major limitations should be considered when interpreting the results of this study: First, the relatively low number of KSMM+ patients. We were able to enroll only seven subjects with MM and this number was too small to address the possible correlation between the extent of the mirror movement and the extent of the changes of the network dynamics. Thus, the precise connection of our findings to the mirror movements in KS remains unclear. Fortunately, even with such a low number, significant (albeit few) differences emerged, suggesting that, by increasing the number of the sample, it will be possible to gain more evidence about this phenomenon. Second, as KS is a disease that mostly affects males, it would be interesting to evaluate what happens to dFC with more female patients at disposal, given that only one was included in our KS sample. Third, little is known, and no data were available, about how the highlighted transient resting-state connectivity states would eventually affect the execution of a motor task. Thus, further work is needed, including the possibility to address this issue by administering motor tasks to the patients. Related to this, an important issue to address would be the choice of window sizes for the sliding-window dFC analysis. Sakoglu et al. (2010) reported that only an ideal window size should be able to estimate dFC variability (capturing the low frequency modes of interest in the rs-fMRI signal) and concurrently detect short-term task-related effects. In this study, functional dynamics were estimated using a validated fixed sliding-window of 150 volumes (about 100s), a measure considered more than reasonable for a 20-min scan, to robustly capture at least two state and the corresponding transition counts. Nonetheless, when attempting to address the influence of the state on the motor response, it is likely that a trade-off existed between the sensitivity for detecting potentially interesting transients in dFC and the signal-to-noise ratio of the task-related FC. Future work should thus evaluate changes across several window lengths that would be then combined in multi-scale approach, e.g., using wavelet transform (Chang and Glover, 2010; Billings et al., 2018). It remains, that the peculiar phenomenon of MM in KS seems to be a good pathological model to investigate spectrally selective variations in long resting-state fMRI sessions and further studies will possibly confirm or better explain the highlighted dynamics behind the pathogenic hypothesis of MM.

## Data availability statement

The raw data supporting the conclusions of this article will be made available by the authors, without undue reservation.

## Ethics statement

The studies involving human participants were reviewed and approved by Ethical Committee at the University Hospital “San Giovanni Di Dio e Ruggi D’Aragona” of Salerno. The patients/participants provided their written informed consent to participate in this study.

## Author contributions

FE, RM, AS, and GV contributed to conception and design of the study. GV organized the database. FD performed the statistical analysis and wrote the first draft of the manuscript. AC and FE wrote sections of the manuscript. All authors contributed to manuscript revision and read and approved the submitted version.

## Footnotes

1
www.brainvoyager.com

2
http://rfmri.org/DPARSF

3
http://www.fil.ion.ucl.ac.uk/spm

4
http://rfmri.org/DPABI

5
https://github.com/trendscenter/gift

6
www.themathworks.com
